# The role of hormones in parasitic plant infection

**DOI:** 10.1093/pcp/pcaf132

**Published:** 2025-10-14

**Authors:** Anna K van Wüllen, Martina Leso, Brikti Ferede Hailu, Kirsten Krause, Charles W Melnyk

**Affiliations:** Department of Plant Biology, Linnean Center for Plant Biology, Swedish University of Agricultural Sciences, Almas allé 5, 756 51 Uppsala, Sweden; Department of Plant Biology, Linnean Center for Plant Biology, Swedish University of Agricultural Sciences, Almas allé 5, 756 51 Uppsala, Sweden; Department of Arctic and Marine Biology, UiT The Arctic University of Tromsø, 9037 Tromsø, Norway; Department of Arctic and Marine Biology, UiT The Arctic University of Tromsø, 9037 Tromsø, Norway; Department of Plant Biology, Linnean Center for Plant Biology, Swedish University of Agricultural Sciences, Almas allé 5, 756 51 Uppsala, Sweden

**Keywords:** hormones, auxin, cytokinin, parasitic plants, haustoria

## Abstract

Plant parasitism is a widespread lifestyle found throughout the plant kingdom that plays important roles in ecology and agriculture. Parasitic plants rely on the formation of specialized parasitic organs called haustoria to invade their hosts and withdraw nutrients. Currently, our knowledge is growing regarding how parasitic plants use haustoria to infect their hosts, modify their physiology and regulate infection. Important factors in plant development are hormone signaling molecules that play essential roles in how plants grow and interact with their surroundings. In recent years, major progress has been made in understanding the relevance of various hormones in plant parasitism. Here, we review recent findings in the field, focusing on the role of hormones in several stages of parasitism, including haustoria induction, vascular development, and interaction with the host. We discuss and compare how hormones influence haustoria development in different parasitic plant lifestyles and species, and identify knowledge gaps in the field. Future work on understanding how hormones influence parasitism is crucial to develop novel ways to control the damage caused by parasitic plants to agriculture, and to discover how parasitic plants efficiently connect to their hosts.

## Introduction

Parasitic plants are a diverse group of plants that attach to and feed on other plants, referred to as hosts. The parasitic lifestyle has emerged at least 12 independent times during plant evolution in the eudicot and magnoliids clades (Nickrent 2020), resulting in over 4000 species of parasitic plants with different parasitic strategies. Some parasitic plants rely entirely on their hosts for survival (obligate parasites), while others can complete their life cycle in the absence of a host (facultative parasites). Some parasitic plants, called hemiparasites, retain the ability to photosynthesize and are independent of the hosts’ sugars. Holoparasites, on the other hand, rely on their host as the source of fixed carbon (Westwood et al. 2010). Finally, parasitic plants infect either the host roots or host stems, but apparently never both. Despite their differences, all parasitic plant species develop a common infective structure to penetrate and establish vascular connections with their host: the haustorium. Haustorium development can be summarized in four main steps. First, the presence of the host is perceived by parasitic plants through host-derived chemicals or signals. Certain parasitic plant species germinate in response to host-derived compounds, whereas all parasites initiate the infection process using host-derived mechanical, light, or chemical signals, also known as haustorium-inducing factors (HIFs), which are often specific to the species of parasitic plant (Goyet et al. 2019). Next, the parasite tissues at the site of host contact expand and elongate toward the host to form an initial swelling called the prehaustorium (O'Malley and Lynn 2000). When the prehaustorium reaches the host tissues, it attaches and the process of host penetration starts. Host penetration relies on the loosening of the host cell walls so that the haustorium can infiltrate between or through the host cells and reach the host vasculature (Losner-Goshen et al. 1998, Olsen and Krause 2017, Auriac et al. 2024, Edema et al. 2024, Leso et al. 2024). Finally, a vascular connection develops in the haustorium to connect the parasite with the host. Holoparasites, being dependent on host-derived organic nutrients, need to connect to both phloem and xylem strands, while for fully photosynthetic hemiparasites like mistletoe, the differentiation of only xylem strands has been described (Irving and Cameron 2009). Once the vascular connection is completed, the parasite can withdraw nutrients and water from the host. Additionally, host and parasite exchange molecules including proteins, RNAs, and hormones suggesting a bidirectional information flow (Spallek et al. 2017, Shahid et al. 2018).

Plant hormones are small mobile signaling molecules that control virtually every step of plant growth, development, and response to their surroundings. They can be synthesized by most plant organs and are transported locally through symplasmic transport, apoplastic transport, or systemically via the vasculature. Classical plant hormones include auxin, cytokinin, gibberellic acid (GA), ethylene, abscisic acid (ABA), jasmonic acid (JA), and salicylic acid (SA). More recently, other hormones have been identified including brassinosteroids (BRs), strigolactones (SLs), and small mobile signaling peptides (Wang and Irving 2011). Given their central role in plant development and environmental response, it is plausible that many of these hormones could play roles in both haustorium formation and host–parasite interactions. In recent years, research has focused on hormonal signaling during plant parasitism to understand haustorium formation and development, and as a possible means to regulate parasitic plant infections in agriculture. While auxin, cytokinin, and SLs have been widely studied and characterized in several parasitic plant species, our knowledge on other hormones like GA, ABA, BR, and peptide hormones is still very fragmented and limited. In particular, exciting and recent research in the field of small peptides including CLAVATA3/EMBRYO SURROUNDING REGION (CLE), TRACHEARY ELEMENT DIFFERENTIATION INHIBITORY FACTOR (TDIF), TDIF RECEPTOR (TDR), GLYCOGEN SYNTHASE KINASE3 (GSK3), and ROOT MERISTEM GROWTH FACTOR (RGF) indicate that these molecules play previously overlooked but important roles during haustoria initiation and morphogenesis. Several recent reviews have laid foundational insights into hormone-driven organogenesis and host–parasite interactions (Cui et al. 2023, Kirschner et al. 2023), including comparing the role of hormones in different symbiosis-driven processes (Jhu et al. 2025). Here, we instead comprehensively review current knowledge on the role of multiple hormones in different parasitic plants, with a focus on haustorium induction and development. We provide an up-to-date overview of the field, including a detailed breakdown of classic hormones and insights into rapidly developing research areas such as peptide signaling and hormone–peptide crosstalk. We also discuss more broadly the roles of hormones in parasitic plant-host interactions.

## Auxin

The phytohormone auxin plays a critical role in parasitic plant infection and controls both early and late stages of infection. In obligate root parasitic plant species, a radicle elongates towards the host plant after germination. In seeds of *Orobanche minor* and *Striga hermonthica* radicle growth is inhibited by accumulation of indole-3-acetic acid (IAA) auxin, either by blocking efflux or through auxin treatments (Kuruma et al. 2021, Tsuzuki et al. 2024). Auxin receptor antagonists like auxinole can rescue the radicle growth inhibition phenotype, suggesting a critical role for auxin in radicle elongation. The polarity of auxin signaling appears important for bending toward the host rather than for the elongation itself (Tsuzuki et al. 2024).

After radicle elongation, the prehaustorium develops. The differentiation from radicle to prehaustorium in *S. hermonthica* is associated with the reduction in the meristematic activity of the root-tip and the downregulation of stem cell genes (*PLETHORA1*, *CYCLINB1*, and *HISTONE4*) (Xiao et al. 2022). The PIN-FORMED proteins (PIN1 and PIN2) change localization to basal polarity in the epidermis, causing the export of auxin and reducing auxin levels in the root tip. This auxin reduction is thought to arrest cell division and stop meristematic activity, allowing the differentiation of the prehaustorium. An auxin biosynthesis inhibitor (l-kynurenine) increases the number of prehaustoria, while treatments with the polar auxin transport inhibitor *N*-1-naphthylphthalamic acid (NPA) reduce prehaustoria formation, consistent with auxin reduction by polar auxin transport being important for successful transition from radicle to prehaustorium stage (Xiao et al. 2022). Disrupted auxin transport and activity also reduces the number of mature haustoria in different parasitic plants like *Phelipanche aegyptiaca* and *S. hermonthica* (Bar-Nun et al. 2008, Xiao et al. 2022, Tsuzuki et al. 2024). Therefore, auxin and auxin efflux appear to play an essential role during the early stages of radicle elongation and haustoria development in obligate root parasites.

**Figure 1:**
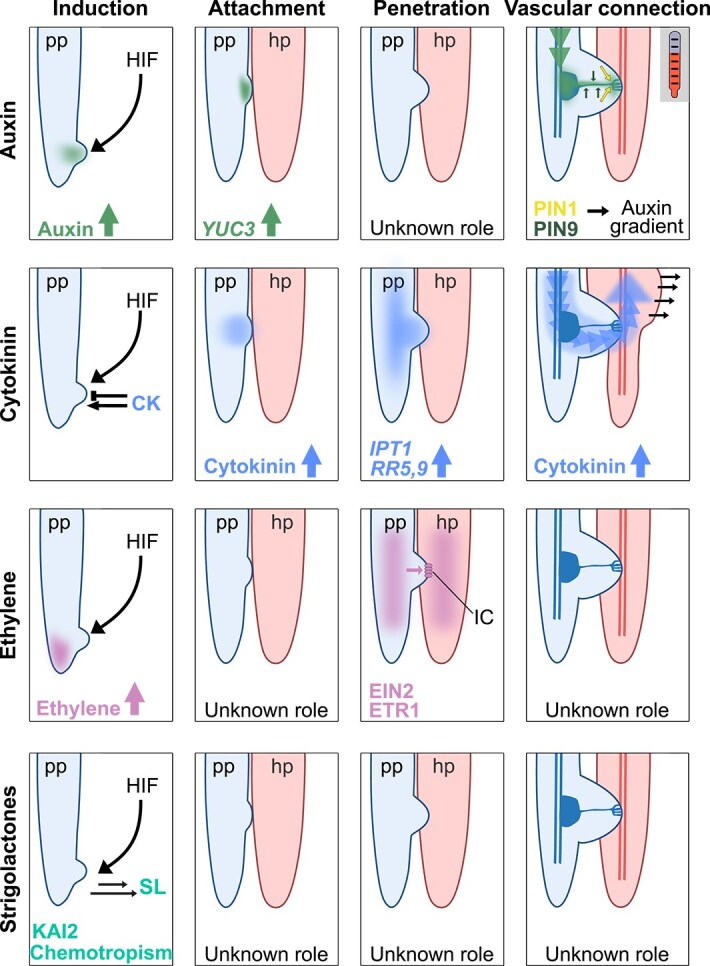
The role of plant hormones during the infection by a parasitic plant forming a root lateral haustorium. Auxin: Accumulation of auxin in the prehaustorium initiation site is caused by haustoria inducing factors (HIFs). During attachment, the auxin biosynthesis gene *YUC3* is expressed in the parasite at the site of host contact. The role of auxin during penetration is unknown. During vascular connection, PIN proteins create an auxin gradient that drives xylem bridge formation connecting the parasite and host vasculature. This process is enhanced by elevated temperatures. Cytokinin (CK): Exogenously applied cytokinin can promote haustoria induction in some species while inhibit haustoria induction in others. The role of cytokinin during attachment is unknown. During the penetration phase, host roots trigger the upregulation of the cytokinin biosynthesis gene *IPT1* and cytokinin responsive genes such as *RR5* and *RR9*. During vascular connection, parasite derived cytokinin triggers host tissue expansion (hypertrophy). Ethylene: HIFs induce the accumulation of ethylene in the root tip of the parasite. The role of ethylene during attachment is unknown. During the penetration process, ethylene-related ETR1 and EIN2 in parasite and host are important for infection. Ethylene signaling in both host and parasitic plant is also important for successful haustoria invasion and the transition of the apex into intrusive cells (IC). During vascular connection, the role of ethylene is unknown. Strigolactones (SL): Strigolactones can act as chemoattractants to guide the parasite to the host via the KAI2 receptor. The role of strigolactones during prehaustoria formation, attachment, penetration and vascular connection is unknown. pp: parasitic plant; hp: host plant.

In other parasitic plant species, auxin has a positive effect on prehaustoria formation. *Triphysaria versicolor* accumulates auxin at the root tip when exposed to HIFs and the application of an auxin transport inhibitor at the root tip induces the formation of prehaustoria-like structures, presumably by mediating auxin accumulation in the root tip where cells are unable to transport the auxin any further (Tomilov et al. 2005) (Fig. 1). Early stages of prehaustoria formation in the stem parasite *Cuscuta campestris* show enhanced auxin transport and auxin response, while tissues of the infection site in *Cuscuta reflexa* and the host *Solanum lycopersicum* show enhanced auxin levels that might be responsible for the morphological changes during the infection process since the artificial injection of IAA could mimic those morphological changes (Löffler et al. 1999, Jhu et al. 2022). In the hemiparasite *Phtheirospermum japonicum*, the exogenous application of the synthetic auxin 2,4-d increases prehaustoria numbers in the presence of the HIF 2,6-dimethoxy-*p*-benzoquinone (DMBQ) (Aoki et al. 2022). Furthermore, in *P. japonicum*, the auxin biosynthesis gene *YUCCA3* (*PjYUC3*) is highly induced by host exudates and upon contact of host and parasitic plant during prehaustoria initiation (Ishida et al. 2016) (Fig. 1). *PjYUC3* expression forms local auxin maxima and knocking down *PjYUC3* expression results in fewer haustoria during infection compared to control *P. japonicum* roots. The ectopic expression of YUC3 at the epidermis of the root leads to the induction of prehaustorium-like structures (Ishida et al. 2016). These findings support the importance of auxin biosynthesis genes like *YUCCAs* that are likely inducing auxin biosynthesis upon HIF recognition resulting in accumulation of auxin and formation of haustoria (Fig. 1).

The parasite connects to the host vasculature through the formation of masses of tracheary elements near the parasite’s vasculature, termed plate xylem, and through the formation of xylem strands connecting parasite xylem to host xylem, termed xylem bridges. Auxin transport plays a crucial role in the differentiation of these vascular elements (Fig. 1) (Wakatake et al. 2020). The auxin efflux inhibitor NPA prevents xylem bridge formation in *P. japonicum*, but does not alter plate xylem (Wakatake et al. 2020). The auxin influx inhibitor 4-hydroxyphenylacetic acid (CHPAA) alters morphology of plate xylem and xylem bridge structure, highlighting the auxin network that underlies xylem bridge formation (Wakatake et al. 2020). The auxin influx transporters AUXIN1/LIKE AUXIN1 (AUX1/LAX) and the polar localization of the auxin efflux transporters PIN1 and PIN9 are important to generate a gradient of auxin to initiate xylem bridge connection. Both *PIN1* and *PIN9* knockdown in *P. japonicum* roots have fewer successful xylem connections. Auxin biosynthesis inhibitors l-kynurenine and yucasin partially block or delay xylem bridge formation when applied after haustorium initiation, but do not fully inhibit it (Wakatake et al. 2020). The effect of temperature upon xylem bridge formation in *P. japonicum* is dependent on auxin transport in the leaves (Serivichyaswat et al. 2022). Elevated temperatures cause accelerated xylem bridge formation in *P. japonicum* and this enhancement can be blocked by application of NPA to the parasite leaves, suggesting leaf-derived auxin is transported to the haustorium in a temperature dependent manner influencing formation of xylem connection between host and parasitic plant (Serivichyaswat et al. 2022). In *S. hermonthica*, auxin biosynthesis and transport genes like *YUCCAs* and *PINs* are differently expressed during haustoria formation and a close relationship between auxin and nitrogen has been demonstrated (Kokla et al. 2022). While nitrogen reduces the number of haustoria, exogenously applied auxin rescues the effect in *S. hermonthica*. However, auxin does not overcome the inhibitory effect of nitrogen in *P. japonicum*, suggesting a species specific regulation and interaction of auxin with nitrogen signaling (Kokla et al. 2022). To conclude, auxin plays an important role across parasitic plants in promoting multiple stages of haustoria development. Its precise regulation through biosynthesis, influx, efflux, and the dependency on PIN transporters is essential for successful infection by parasitic plants.

## Cytokinin

Much like auxin, cytokinin plays multiple roles in parasitic plants. In the stem parasite *Cuscuta japonica*, blue light induces twining whereas far red light causes prehaustoria induction. In the absence of red/far red light, cytokinin induces prehaustoria formation when combined with blue light, whereas cytokinin and brassinosteroid treatment together induce prehaustoria in the dark (Furuhashi et al. 2021). These data suggest that cytokinin signaling promotes prehaustoria induction and points to a coordination of environmental and hormonal cues to regulate haustoria.

Cytokinins from the host can also act as HIFs in many root parasitic plant species, including the facultative root parasite *T. versicolor* and the obligate root parasites *Phelipanche ramosa*, *S. hermonthica*, and *Striga asiatica* (Keyes et al. 2000, Wrobel and Yoder 2001, Goyet et al. 2017, Aoki et al. 2022). In *P. ramosa*, host root exudates trigger prehaustorium development and induce the cytokinin responsive genes *RESPONSE REGULATOR 5* (*PrRR5*), *CYTOKININ OXIDASE 2* (*PrCKX2*) and *4* (*PrCKX4*), and *ZINC FINGER PROTEIN 6* (*PrZFP6*). Similarly to host exudates, the exogenous application of different cytokinins triggers the development of prehaustorium-like structures, although a molecular validation of the prehaustoria identity of these structures is still lacking (Goyet et al. 2017). Co-application of the cytokinin signaling inhibitor PI-55 with exogenous cytokinin reduces the effects of exogenous cytokinin (Goyet et al. 2017). In *S. hermonthica*, prehaustoria induction by exogenous cytokinin depends on cytokinin perception by the HISTIDINE KINASE (AHK) receptors, since treatment with LGR-991, a CK-analog that prevents CKs from binding AHK receptors, prevents prehaustoria formation in the presence of exogenous cytokinin. LGR-991 treatment does not inhibit prehaustoria induction by DMBQ, a quinone HIF, suggesting DMBQ-mediated prehaustoria induction is independent of cytokinin perception (Aoki et al. 2022). However, treatment with the DMBQ-analog TFBQ inhibits both DMBQ- and CK-induced prehaustoria development, suggesting CKs act through a common downstream pathway with DMBQ, possibly through *ShPIRIN* and *ShYUC3* (Aoki et al. 2022). However, most of these experiments used exogenous cytokinin applications to induce prehaustoria and the role of endogenous cytokinins in host root exudates needs further investigation.

In addition to cytokinins released through host root exudates, cytokinins are also synthesized by parasitic plants during infection. Cytokinin levels increase in *Santalum album* haustoria (Zhang et al. 2012), as well as in the haustoria of *C. japonica* and *P. japonicum* (Furuhashi et al. 2014, Spallek et al. 2017), suggesting a role for parasite-derived cytokinins in infection. Cytokinin is not a HIF in the facultative root parasite *P. japonicum* (Aoki et al. 2022). However, prehaustoria development in *P. japonicum* induces cytokinin accumulation and an increased expression of cytokinin-responsive genes like *PjRR5* and *PjRR9* both locally at the site of haustoria formation and systemically (Spallek et al. 2017, Kokla et al. 2025) (Fig. 1). Exogenous application of cytokinin inhibits the formation of haustoria both locally and systemically in *P. japonicum* (Kokla et al. 2025), suggesting cytokinin acts as a regulator of haustoria numbers in some parasitic plant species. Furthermore, increasing cytokinin degradation in the parasite by overexpressing the cytokinin oxidase *AtCKX3* increases haustoria numbers formed by *P. japonicum*, consistent with cytokinin’s role as a haustorium inhibiting factor. Such an inhibitory signal appears highly relevant for long distance negative regulation of haustoria by existing haustoria, a phenomenon also observed in another facultative root parasite, *Parentucellia viscosa* (Kokla et al. 2025).

Cytokinins are also important for the interaction between parasitic plants and their hosts. Cytokinins produced by *P. japonicum* move into the *Arabidopsis thaliana* host to trigger tissue growth in host roots above the haustoria which might facilitate nutrient acquisition by the parasite (Spallek et al. 2017). Such hypertrophy is dependent on cytokinin accumulation and signaling in the host (Spallek et al. 2017) (Fig. 1). *Arabidopsis thaliana* mutants impaired in cytokinin biosynthesis still show hypertrophy phenotypes, indicating that hypertrophy is not caused by cytokinin biosynthesis in the host, while overexpressing *AtCKX3* in the parasite is sufficient to inhibit host hypertrophy (Spallek et al. 2017). *P. japonicum* hairy roots mutated in the cytokinin biosynthesis gene *ISOPENTENYLTRANSFERASE1a* (*PjIPT1a*) do not induce root hypertrophy in the host (Greifenhagen et al. 2021), confirming a role for parasite-derived cytokinin. Homologs for *PjIPT1a* are also found in the obligate parasites *S. hermonthica* (*ShIPT1a*) (Greifenhagen et al. 2021) and *S. album* (*SaIPT1*) (Zhang et al. 2015), where they are also upregulated during infection, suggesting multiple species of parasitic plants might utilize similar pathways to modify host development and facilitate nutrient acquisition. The hypertrophy of host tissues in particular is a widely utilized strategy in plant parasitism, also shared by mistletoe (*Viscum album*), *C. japonica*, and *Alectra vogelii* (Heide-Jørgensen 2008, Furuhashi et al. 2014, Spallek et al. 2017). However, research is needed to test the role of cytokinin in hypertrophy induction in these species.

## Ethylene

Ethylene signaling plays an important role both in the parasitic plant and in the host during different stages of the infection process. Early reports found that in *S. hermonthica*, gaseous ethylene induces germination and an inhibitor of ethylene biosynthesis inhibited germination (Bebawi and Eplee 1986, Logan and Stewart 1991). In *T. versicolor*, treatment with HIFs induces the expression of ethylene-inducible promoters, and the accumulation of ethylene in the root tip has an important role in lateral prehaustoria development (Fig. 1) (Tomilov et al. 2005). Applying the ethylene precursor 1-aminocyclopropane-1-carboxylate (ACC) enhances prehaustoria formation in *T. versicolor* in the presence of DMBQ, whereas blocking ethylene biosynthesis or signaling reduces prehaustoria formation (Tomilov et al. 2005) consistent with ethylene promoting prehaustoria in *T. versicolor.* However, in *P. japonicum*, blocking ethylene signaling has no effect on prehaustoria formation and mutations in ethylene signaling genes *ETHYLENE RECEPTOR1* (*ETR1*) and *ETHYLENE INSENSITIVE PROTEIN2* (*EIN2*) have little effect on prehaustoria formation (Cui et al. 2020). These findings suggest that ethylene signaling is not essential for prehaustoria formation in *P. japonicum* and that the requirements for ethylene signaling in prehaustoria initiation vary between species. During host invasion, *ETR1* and *EIN2* play important roles in *P. japonicum*, and the respective mutants in *P. japonicum* are unable to differentiate haustorial apex cells into intrusive cells resulting in severe defects in host invasion (Cui et al. 2020) (Fig. 1). Furthermore, disturbing ethylene signaling in the host plant also reduces penetration. *Arabidopsis etr1* and *ein2* mutants, as well as a mutant lacking seven isoforms of the ethylene biosynthesis genes *ACC SYNTHASE (ACS)*, get less often invaded indicating that ethylene biosynthesis and perception in the host also play important roles during the haustoria invasion of *P. japonicum* (Cui et al. 2020). Arabidopsis *ein2* did not show altered infection rates with *P. aegyptiaca* but an *ETHYLENE RESPONSE FACTOR1* (*AtERF1*) overexpressing line that acts downstream of ethylene signaling was significantly less susceptible to *P. aegyptiaca* attachment (Lorenzo et al. 2003, Clarke et al. 2020). The parasitic plant *C. campestris* also relies on the host’s ethylene signaling during haustoria formation (Narukawa et al. 2021). When *C. campestris* invades its host, epidermal cells of the haustorium differentiate into elongated finger-like cells called search hyphae, which find and connect with the host vasculature. Upon infection, *ACS2* and *ACS6* are upregulated in infected host tissues. The respective host Arabidopsis *acs* octuple mutant with defects in ethylene biosynthesis shows a reduction in search hyphae elongation, which can be complemented by ACC, consistent with the importance of ACC biosynthesis for hyphae elongation (Narukawa et al. 2021). On the other hand, other Arabidopsis ethylene mutants like *etr1* do not show the same phenotype, indicating that only specific ethylene signaling pathway components are involved and important for successful *C. campestris* invasion (Narukawa et al. 2021). Ethylene seems to be an important factor in the infection of parasitic plants where both parasite-derived and host-derived ethylene are relevant and the disruption of either leads to an altered infection phenotype.

## Strigolactones

Many studies have investigated the role of SLs during the infection by root parasitic plants, making them perhaps the best-studied phytohormone in the context of parasite–host interactions. Their main significance in the context of root parasitic plants lies in SLs acting as germination stimulants and chemoattractants, giving away not merely that a host is present but also indicating its location relative to the parasite. Consequently, the group of parasites that has been in the limelight of SL research is root parasites of the Orobanchaceae family (Bouwmeester et al. 2021). It was recently demonstrated that SL biosynthesis genes are conserved in root parasites (Okawa et al. 2025). What role these endogenous SLs play for the (pre)haustorium development and the further interaction with the hosts has yet to be investigated. Below, we will thus focus only on the effects host-derived SLs have on root-parasitic plant infections.

SL biosynthesis and regulation have been covered in several comprehensive recent reviews (Jia et al. 2018, Nelson 2021). Here, we highlight that SLs provide an excellent biochemical compass for the parasites to identify promising hosts in their vicinity (Fig. 1). Many host plants secrete SLs into the rhizosphere to attract mycorrhizal symbionts, particularly under nutrient and phosphate limiting conditions. Such synthesis and exudation are thus highly dependent on the nutrient status of the hosts (Bouwmeester et al. 2007). More than 35 SLs have been identified in different plant species and a single plant species can exude mixes of SLs in different combinations from their roots (Yoneyama et al. 2010). The biological activities of SLs and, consequently, their influence on parasitic plant–host plant interaction vary strongly with this structural diversity (Dun et al. 2023), providing opportunities for parasites to co-evolve with their preferred hosts. Biochemical and genetic evidence from *S. hermonthica* demonstrated that highly sensitive receptor proteins have indeed evolved in the parasite by neofunctionalization from nonparasitic plant receptors mediating responses to SLs and the structurally related karrikins (Nelson 2021). Parasitic plant paralogs of KARRIKIN INSENSITIVE 2 (KAI2)/HYPOSENSITIVE TO LIGHT have a unique ability of binding various host-derived SLs and are among the key players in SL-mediated parasitic seed germination in Orobanchaceae (Tsuchiya et al. 2018, Kee et al. 2023). These receptor proteins, however, trigger different responses in parasitic plants. Manipulating the genes that are involved in detection and biosynthesis of SLs is a promising strategy to reduce the severity of parasitic weed attacks, while maintaining the essential function of SLs in host plant development (En-nahli et al. 2023). *KAI2* clade genes are also conserved in *P. japonicum* although this parasite does not require exogenous SL for germination (Takei et al. 2024), unlike obligate parasitic species of the Orobanchaceae family. Interestingly, strigol stimulated *P. japonicum* germination under nitrate-restricted conditions, but not in the presence of nitrate, indicating that in nitrate-poor soils, the presence of a host can override nitrate-dependent germination inhibition. However, the effect was weak (Ogawa and Shirasu 2022).

*P. japonicum* and *S. hermonthica* use SLs as chemoattractants, guiding their roots toward those of their host plants (Fig. 1). The chemotropic effect of compounds such as rac-strigol, rac-orobanchol, and synthetic analogs like GR24 might have evolved exclusively in parasitic species since nonparasitic plants do not exhibit this response (Ogawa et al. 2022). Recent research has found that SLs trigger the development of prehaustorium-like structures on the facultative hemiparasitic plant *Castilleja* (Orobanchaceae) (Burger et al. 2024). Multiple KAI2d receptor proteins, particularly KAI2d15, were identified as potential SL receptors in *Castilleja*. These receptors facilitate the plant’s ability to detect SLs and initiate prehaustorium formation. Altogether, SLs play critical roles in early stages of host identification but whether they also play a role in later stages of infection remains unknown (Fig. 1).

In *Cuscuta*, SLs do not appear to play a role during the infection process itself. While recent studies provided circumstantial evidence for SLs being mobile elements in dodder-mediated interplant communications (Zhao et al. 2025, Zheng et al. 2025), they did not reveal an effect on the parasite itself. For more comprehensive conclusions, quantitative and qualitative studies of haustorium formation on hosts containing mutations in the SL biosynthetic pathway would be needed and should be a future goal.

## Peptide Hormones

Peptides play an important role in plants to regulate various developmental processes and a range of peptides were identified that regulate parasitic plant processes during attachment and infection.

A group of microRNA-encoded peptides (miPEPs) affect parasitic plant germination. MicroRNAs are important small RNAs that cleave mRNA targets to negatively regulate transcription (Tourneur et al. 2025). Certain miRNAs encode and produce miPEPs that can activate their own transcription in a positive feedback loop and thereby downregulate mRNA targets. In *Orobanche cumana*, 11 miPEPs were identified and the regulation of their microRNA targets affects radicle elongation. MiPEP319a strongly inhibits germination through downregulation of the corresponding *TEOSINTE BRANCHED1/CYCLOIDEA/PROLIFERATING CELL FACTOR* (*TCP*) and *MYELOBLASTOSIS* (*MYB*) target genes of miRNA319 (Tourneur et al. 2025).

In *C. japonica*, a peptide signaling network is activated in the haustoria mediated by TDIF, TDR, and GSK3 (Shimizu et al. 2018). In *A. thaliana,* the TDIF–TDR–GSK3 network regulates the maintenance of stem cells and proliferation of vascular cambium cells. TDIFs are encoded by small, secreted peptides like CLAVATA3/EMBRYO SURROUNDING REGION41 (CLE41) that bind to the TDRs, which activate *WUSCHEL RELATED HOMEOBOX4* (*WOX4*) and *GSK3*. *WOX4* maintains stem cells and *GSK3* inhibits the differentiation into xylem vessels through negative regulation of BRI1-EMS-SUPPRESSOR1 (BES1). In *C. japonica* players of this network are expressed after host attachment including *GSK3*, *WOX4*, *BES1*, and *CLE41* (Shimizu et al. 2018). Since the expression of *WOX4* and *CLE41* is almost synchronized, it is plausible there is another CLE or different peptide that regulates *TDIFs* and *WOX4* expression. Another possibility would be that the core machinery in *C. japonica* is activated by a host TDIF. Interestingly, an antisense transcript of *CLE41* was found in *Cuscuta* but no interaction or connection between the antisense and the sense expression of *CLE41* was found, thus, it still needs to be shown whether the antisense *CLE41* has a role in downregulating *CLE41* (Shimizu et al. 2018). Fine-tuned expression of the genes around the TDIF–TDR–GSK3 network controls a balance between the WOX4 mediated and GSK3 mediated pathway which leads to the formation of xylem vessels to connect the parasite to the host xylem.

CLEs and other peptides are synthesized as prepropeptides and cleaved into their mature active forms by subtilisin­type proteases (SBT) (Schaller 2004). *P. japonicum* has 14 CLEs and CLE1 is activated by SBT1.2.3 in mature haustoria to form secondary haustoria following the first mature haustoria, suggesting CLE1 plays a role in promoting haustoria formation (Greifenhagen et al. 2024). The application of CLE1 and quinone HIF DMBQ together enhances the number of haustoria, even in the absence of a host, suggesting CLEs and certain HIFs have independent roles in promoting haustoria. Interestingly, *YUC3* expression is also induced by CLE1 at similar levels as by DMBQ, indicating a cross talk between peptide hormones activating auxin-based hormone signaling. CLE1 clusters together with CLE homologs known to regulate the number of nitrogen-fixing nodules in legumes, suggesting that the regulation of the number of secondary haustoria in *P. japonicum* and the regulation of the number of nodules in legumes could have similar molecular mechanisms (Greifenhagen et al. 2024).

A recent study showed that RGF peptides, also known as CLE-LIKE peptides, are upregulated upon HIF treatment, and RGF treatment induces prehaustoria formation in *P. japonicum* and *S. hermonthica*. RGF1, RGF2, and RGF5 induce the expression of the prehaustoria formation initiator *YUC3* in *P. japonicum* linking peptide signaling to auxin signaling (Fishman et al. 2025). Knockouts of the receptors of RGF2 and RGF5 in *P. japonicum* reduce prehaustoria formation. RGF2 seems to be conserved among parasitic Orobanchaceae species and unraveling the mechanisms that induce *RGF2* expression in parasitic and non-parasitic lineages could help to shed light on parasitism evolution (Fishman et al. 2025).

Another conserved peptide is the prepropeptide of CUSCUTAIN, which acts as an inhibitory precursor of the cysteine proteinase. It is expressed in early phases of infection in both *C. reflexa* and *P. aegyptiaca* (Bleischwitz et al. 2010, Rehker et al. 2012). The overexpression of the prepropeptide of *CUSCUTAIN* in *C. reflexa* and its respective homolog in *P. aegyptiaca* results in less infections, suggesting a conserved mechanism for certain parasitic plant peptides and their role in infection processes, despite independent development of parasitism between these two species of parasites (Bleischwitz et al. 2010, Rehker et al. 2012).

Thus, peptide hormones play an important role in haustoria development and regulation, while further research in this emerging field will help identify additional peptides and help better characterize known ones during haustoria formation.

## Abscisic Acid

ABA has an important role in plant development. Examples of ABA-regulated processes include seed dormancy and germination, lateral root growth, and vascular differentiation (Brookbank et al. 2021). In addition to plant growth and development, ABA signaling is also important for plant response and resistance to abiotic stresses, like drought and salinity stress (Zhu 2002).

Several parasitic plant species have reduced sensitivity to ABA. The dodder *Cuscuta australis* shows reduced growth only when concentrations of 700 μM of ABA are applied and germination is only partially delayed when applying 10 μM ABA, compared to tomato where 1 μM ABA which is sufficient to inhibit both growth and germination (Li et al. 2015). This phenotype is likely linked to the contraction of the ABA PIR1-LIKE (PYL) receptors gene family in *C. australis* leading to reduced ABA detection. ABA controls plant transpiration by inducing stomatal closure, and therefore reducing water transpiration and increasing tolerance to stresses like drought (Bharath et al. 2021). Li et al. (2015) introduced the possibility that the reduced ABA signaling in *C. australis* could be connected to a decrease in transpiration, as *Cuscuta* plants show reduced water loss during air drying compared to tomato (Li et al. 2015). However, since stomata in *Cuscuta*, if present at all, are often playing other roles than regulating gas exchange (Clayson et al. 2014), it is likely that reduced ABA signaling has other reasons and consequences than transpiration.

*S. hermonthica* is also known to maintain stomatal aperture even in high drought conditions (Inoue et al. 2013). This phenotype is caused by a mutation in a PROTEIN PHOSPHATASE 2C (*ShPP2C1*), which leads to the suppression of ABA signaling (Fujioka et al. 2019). The increase in transpiration caused by a reduced ABA sensitivity likely generates a water potential gradient that increases the parasites’ sink strength (Inoue et al. 2013). This strategy has likely evolved to facilitate nutrient assimilation from the host through the haustoria vasculature. Furthermore, a reduced sensitivity to ABA might also aid parasitic plants in counteracting the host’s ABA-mediated defense signaling, and maintain their sink strength when the host responds to the lower water availability resulting from parasitism. Despite *S. hermonthica* being highly resistant to ABA, it was recently discovered that the exudates of germinated *S. hermonthica* seeds contain high ABA levels, which inhibit the germination of other surrounding *S. hermonthica* seeds, and promote host root growth towards the parasitic plant (Jamil et al. 2024).

ABA also appears to be a negative regulator of parasitism in *P. japonicum*. Exogenous ABA application inhibits haustoria development in *P. japonicum*, whereas endogenous ABA levels increase following nitrogen application, suggesting ABA signaling is used by some parasitic plants as a means to suppress haustoria formation in response to high nutrient levels (Kokla et al. 2022). These effects of ABA upon infection are however not observed in *S. hermonthica* (Kokla et al. 2022), suggesting facultative and obligate parasites might adopt different strategies to regulate haustoria numbers in response to nutrient levels, perhaps related to *S. hermonthica*’s high resistance to ABA. ABA signaling has also been linked to nutrient acquisition in dodders. *Cuscuta reflexa* has a higher concentration of ABA compared to its host *Vicia faba*, with the highest concentrations of ABA found in the regions of the stem with developed haustoria (Ihl et al. 1984). The increase in ABA levels was suggested to be linked to the uptake of phloem-transported nutrients by Cuscuta from its host or to the storage of such assimilates (Ihl et al. 1984).

Finally, ABA signaling might play a role in differentiating xylem in haustoria, similar to its role in differentiating xylem in non-parasitic plants (Ramachandran et al. 2021). In *P. japonicum*, inhibiting ABA signaling with the chemical fluridone reduced xylem bridge formation (Kokla et al. 2022); however, the molecular mechanisms by which ABA signaling affects haustoria maturation and xylem bridge development have not yet been described but presumably may be similar to those mechanisms found in non-parasitic plants (Ramachandran et al. 2021).

## Gibberellins

The role of GAs in parasitic plants and their interaction with host plants is limited to studies in only a few parasitic plants. Biochemical analyses revealed that *O. minor* has the ability to synthesize certain GA species, specifically GA_38_, GA_47_, and GA_58_, which are not typically found in its host plant. However, the major GAs in *O. minor* are transported from the host, indicating a dependence on its host-derived GAs (Suzuki et al. 1994). *Cuscuta reflexa* was found to contain similar GA concentrations as comparable autotrophic plants (Ihl et al. 1984) and the first sequenced genomes of two parasitic *Cuscuta* species (Sun et al. 2018, Vogel et al. 2018) revealed that genes from the GA biosynthetic pathway are conserved. Interestingly, GA concentrations in infected host tissue but not in *Cuscuta* tissue at the infection sites were found to be increased, thus creating a gradient whose functional significance is to date unknown. The prehaustorial to haustorial stages in *Cuscuta pentagona* were found to be enriched in transcripts with gene ontology terms for GA biosynthesis and metabolism, with specifically genes for the GA biosynthetic enzymes *GA 2-OXIDASES* and *GA 20-OXIDASES* (*GA2OX8*, *GA20OX1*, *GA20OX2*, and *GA20OX5*) being upregulated (Ranjan et al. 2014). Upregulation of *GA20OX* and *GA20OX2* as well as four *GA2OX* genes coding for an enzyme transiting bioactive to inactive forms of GAs was also observed in the hemiparasite *S. album* in haustorial development (Zhang et al. 2015). Meanwhile, the genome sequences for the root parasite *S. hermonthica* (Yoshida et al. 2019) and the endophytic *Sapria himalayana* (Guo et al. 2023) confirmed the retention of the GA biosynthesis pathway in more parasitic plants. However, in *S. himalayana*, two genes encoding key integrators of GA signaling are lost (Guo et al. 2023) and might be replaced by host-derived mRNAs, tentatively indicating a high degree of adaptive evolution in response to their endophytic lifestyle.

The protein GA-INSENSITIVE DWARF1 (GID1) acts as GA receptor, perceiving active GAs, and triggering GA responses (Ueguchi-Tanaka et al. 2005). Higher GID1 accumulation promotes GA bioactivity that in return stimulates GA-dependent plant seed germination and development (Liu et al. 2015). Interestingly, a recent study proposed an experimental evidence-based model for *S. hermonthica* where the direct influence of GAs on germination was abandoned and replaced by an indirect mode of action via SLs (Yap and Tsuchiya 2023). Another recent study confirmed these findings and implied that GA_3_ accumulating during seed conditioning possibly facilitates germination indirectly by upregulating the expression of the ethylene biosynthesis gene *ShACO1* (Chen et al. 2025). New findings that GA concentrations are regulated during touch-dependent morphogenesis in *Arabidopsis* (Wang et al. 2024), could perhaps link them to haustoriogenesis, but this has yet to be investigated.

## Jasmonic Acid and Salicylic Acid

JA and SA play a major role in plant defense against pathogens. Therefore, these pathways are strong candidates to be activated during plant parasitism. Most of the current knowledge on parasitic plants and JA or SA focus on haustoria attachment and interaction with the host. Treating the host with methyl jasmonate or methyl salicylate increases host resistance to *P. aegyptiaca* (Bar-Nun and Mayer 2008). Treating the host *Trifolium pratense* with inducers of SA-mediated defense, but not with inducers of JA, increases resistance to *O. minor* (Kusumoto et al. 2007), suggesting host resistance might follow different pathways for different host species and/or different parasites. Mutations in host SA or JA signaling also affect infection by *P. aegyptiaca* (Clarke et al. 2020). Furthermore, *C. pentagona* and *C. australis* parasitism induce JA and SA accumulation in their tomato hosts, which activate both hypersensitive-response (HR) based and non-HR-based pathogen resistance pathways (J.B. Runyon et al. 2010b, Yang et al. 2025).

More complex interactions between parasitic plants and their hosts involving JA responses have also been reported. Infection by *C. pentagona* reduces the accumulation of JA but increases SA levels in the host tomato, and reduces host susceptibility to the moth *Spodoptera exigua* (Runyon et al. 2008). In *P. japonicum*, no increase was observed in either SA nor JA levels during infection (Kokla et al. 2022).

Overall, the currently available information on SA and JA suggests that these hormones might have a larger role in host response to parasitic plants, but a minor role in haustoria development. As JA and SA signaling are part of the host defense responses against parasitic plants (J.B. Runyon et al. 2010a), parasitic plants might have evolved to avoid JA and SA accumulation during haustorium development to avoid inducing the host defense responses triggered by haustorium invasion.

## Brassinosteroids

BR signaling has been implicated in plant response to different stresses and in maintaining cell wall homeostasis (Wolf et al. 2014, Manghwar et al. 2022). Therefore, BRs are likely to have some role in plant parasitism. BR treatment has recently been reported to delay prehaustoria induction and xylem bridge development in *P. japonicum*, possibly through the inhibition of cell wall modifying enzymes like PECTIN METHYLESTERASES and PECTIN METHYLESTERASE INHIBITORS (Leso et al. 2024). BRs can also act together with cytokinins to replace the light requirement for prehaustoria formation in *C. japonica* (Furuhashi et al. 2021). It is possible that BR signaling is important to regulate cell wall modifications to differentiate haustorial tissues and to invade host tissues (Losner-Goshen et al. 1998, Edema et al. 2024, Leso et al. 2024).

Treatment of *O. minor* seeds with BRs shortened the conditioning time required for the parasitic plant seeds to germinate in response to host germination stimulants and SLs (Takeuchi et al. 1995). BRs were therefore proposed as possible enhancers of “suicidal germination” treatments, a parasitic plant control strategy where the germination of the parasitic plant is induced in the absence of a suitable host (Zwanenburg et al. 2016). More recently, BR treatments on sunflower seeds were also reported to increase their resistance to *O. cumana* infection. The treatment induces an increased immune response in the host and reduces oxidative stress and cellular damage, which are associated with parasitic plant infection (Zhang et al. 2024). Seed pre-treatment with BRs could therefore be a strategy to control parasitic plant numbers and increase host resistance in the field. However, more research is needed on how BRs could be applied in agricultural practices and the role of BR signaling in host invasion.

## Conclusions and Future Directions

**Figure 2:**
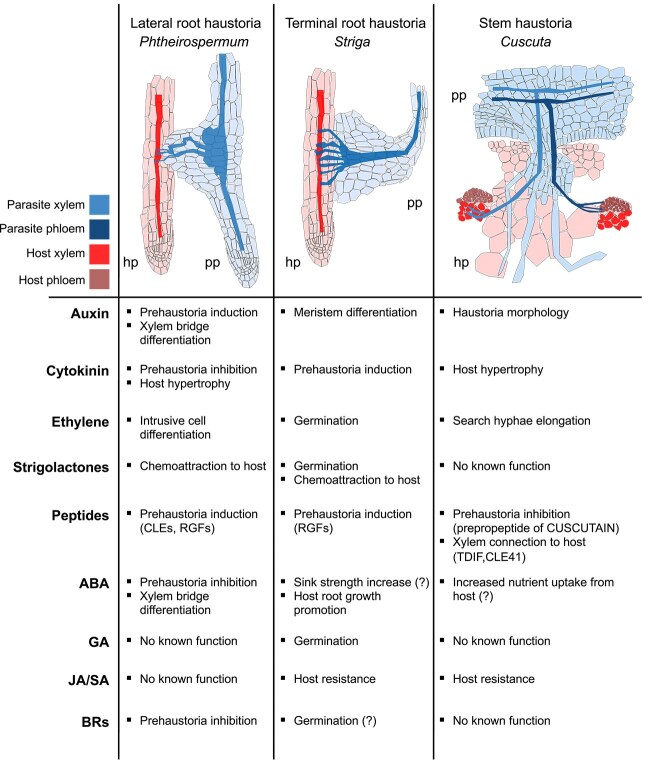
The role of different plant hormones during the formation of diverse haustoria. Examples are presented for *Phtheirospermum japonicum*, *Striga hermonthica*, and the genus *Cuscuta* that form root lateral haustoria, root terminal haustoria, and stem haustoria, respectively. pp: parasitic plant; hp: host plant.

Plant hormones play critical and wide-reaching roles in parasites, hosts, and non-parasites. A major interest lies in how parasites have modified their hormone signaling and hormone response pathways to perceive hosts, form haustoria, and modify host physiology. Key roles have emerged for SL perception, ABA resistance, auxin-induced tissue growth, and the systemic movement of cytokinin. Such roles include developmental roles related to cell division and cell differentiation, but also roles related to promoting or inhibiting germination or haustoria formation based on environmental cues such as host presence or nutrient availability. Hormones have advantages as signaling molecules that are systemically transported between cells, organs, tissues, and between parasite and host. This inter-plant communication likely extends beyond classical hormones and is more broadly thought to encompass mobile mRNAs, sRNAs, and proteins (Kim et al. 2014, Shahid et al. 2018). The roles of multiple hormones have been described through physiology assays, bioinformatics, and heterologous systems (Fig. 2), but significant mechanistic details are lacking. A future priority should focus on the role of hormones in parasites including their perception, signaling, downstream targets, and spatiotemporal regulation. Similarly, peptide hormones such as CLEs and RGFs activate auxin biosynthesis through *YUC3* upregulation in *P. japonicum*, thus linking peptides with canonical hormones. How widespread and relevant this is in other parasite species remains unknown and investigating such cross talk between various hormones, peptides, and RNAs should be another major priority. One significant challenge to achieving these goals is a lack of stably transformed parasitic plants. Recent progress has been made with *C. campestris* (Adhikari et al. 2025, Alles et al. 2025), but this method is challenging to use and presents low success rates. Developing robust and easy transformation techniques that allow heritable CRISPR-Cas9 modifications, reporter transgenes, or overexpression lines would be a critical advance for the study of parasitic plants. In addition, several parasitic plants species are emerging as major players in our understanding of how parasites infect their hosts, for example, *Cuscuta*, *Phtheirospermum*, and *Striga* (Fig. 2). These species benefit from sequenced genomes and well described physiology. Fundamental discoveries in parasitic plant research will likely continue to be made in these species, yet other parasite species models of ecological and agricultural relevance should also be developed and advanced.

Plant hormones serve additional roles by providing insight into parasitic plant evolution. Several plant hormones including ABA, JA, SA, auxins, and SLs originate from precursors that are synthesized in the chloroplasts (Bittner et al. 2022, Bajguz and Piotrowska-Niczyporuk 2023). Given that several parasitic plant species have plastid genome reductions, this may have had implications for the evolution of this organelle in parasitic plants (Krause 2008, Schneider et al. 2018, Wicke and Naumann 2018). For example, the large subgenus *Grammica* of the dodders (genus *Cuscuta*) exhibits high amounts of carotenoids despite having no net photosynthetic carbon fixation. It is possible that such precursors or hormones are sequestered from the host with the general nutrient stream, or more likely, that the parasites have retained control over some, if not all, of their own phytohormones and offering new tentative arguments why the maintenance of the plastid compartment as a separate metabolic space, including the retention of a stripped-down plastome, is mandatory.

In addition to providing developmental and evolutionary insights, plants hormones provide intriguing mechanisms for controlling and regulating parasitism. Major research is focused on identifying stable synthetic germination stimulants that can be deployed to control parasites like *Striga* (Zarban et al. 2022). Similarly, providing fertilizer to nutrient poor fields provides an effective means to partly control the effects of Striga likely by improving host strength, reducing SL production by the host and by reducing haustoria numbers in the parasite (Jamil et al. 2021, Kokla et al. 2022, Shi et al. 2025). Hormones that inhibit or promote haustoria formation such as ethylene, auxin, cytokinin, ABA, or SLs could be modified in the host to suppress haustoria formation. For instance, recent work in tomato showed that resistance to *P. aegyptiaca* was indeed achieved by inhibiting SL exudation in tomato (Ban et al. 2025). Discovering novel signaling compounds that either suppress germination or promote early germination could play important roles in resistance. Similarly, parasitic plants have an unusual ability to fuse their tissues to distantly related plants and better understanding this ability could allow us to engineer plant–plant interactions. We could use this knowledge to promote such interactions, such as improving plant grafting, or use it to inhibit interactions, such as to combat parasitic plants or to block natural root fusions that can transfer disease between plants. Through a combination of fundamental and applied developments, our understanding of plant hormone biology will continue to develop and contribute to a better understanding of the ecological importance of parasitic plants while providing new strategies to mitigate their negative impacts.

## Data Availability

No new data were generated or analyzed in support of this research.
